# Dataset on individual differences in self-reported personality and inferred emotional expression in profile pictures of Italian Facebook users

**DOI:** 10.1016/j.dib.2022.107899

**Published:** 2022-02-05

**Authors:** Davide Marengo, Michele Settanni, Christian Montag

**Affiliations:** a Department of Psychology, University of Turin, Via Verdi 10, Turin 10124, Italy; b Institute of Psychology and Education, Dept. Molecular Psychology, Helmholtzstraße 8/1, Ulm 89081, Germany

**Keywords:** Facebook, Social media, Emotional expression, Image recognition, Personality, Big five, Impulsivity, Sensation seeking

## Abstract

We retrieved the current profile picture of 2234 Italian Facebook users who also answered self-report questionnaires on demographic variables and personality. Data were collected between March and June 2018 using a Facebook web application. Profile pictures consisting of 200 × 200 resolution jpegs were obtained by sending a request via the Facebook Graph API and analyzed using online commercial services allowing for the scoring of facial expressions in image data, namely Microsoft Azure Face API and MEGVII Face++ Detect API. Both services provide emotional expression scores if at least one face is successfully detected in the picture. Using the Microsoft Azure Face API we obtained scores for anger, contempt, disgust, fear, joy, sadness, surprise, and neutrality. Using the MEGVII Face++ Detect API, pictures were scored for the presence of anger, disgust, fear, joy, sadness, and surprise, and neutrality. Higher scores on each emotion refer to a stronger expression of the respective emotion. The dataset presented here consists of data of N =728 Facebook users with a profile picture in which both APIs detected only one face. Regarding self-report data, the dataset includes the following demographic information about the participants: gender and age. The dataset also includes participants’ personality scores based on a short validated assessment of Big Five traits (Ten Item Personality Inventory), and Impulsivity/Sensation Seeking (IMPSS8). A document including the questions administered in the online survey is attached to the dataset. This dataset can be useful to generate insights on the association between demographic variables, including age and gender, and personality (Big Five traits and Impulsivity/Sensation Seeking), and emotional expression as derived from social media pictures. It can be useful for researchers and data scientists who do research in social sciences, in particular *psychoinformatics*, to train models in order to infer personality of users of social media platforms from profile pictures.

## Specifications Table

**Table:** 

Subject	Social and Personality Psychology
Specific subject area	Association between emotional expression in Facebook profile pictures and self-reported Big Five personality and Impulsivity/Sensation Seeking
Type of data	Table
How data were acquired	Self-report data were acquired using an online survey administered via a Facebook web application. Facebook profile pictures were collected using the Rfacebook package for R. Emotion recognition scores were obtained using the Microsoft Azure Face and MEGVII Face++ Detect APIs.
Data format	Raw, Analyzed
Description of data collection	A Facebook web application was created administering self-report questionnaires and collecting participants’ informed consent for accessing profile data. The application also included a Facebook login collecting participants’ consent allowing sharing of Facebook profile data with the researchers. Moreover, informed consent for participation in the online survey was also collected via the application.
Data source location	Country: Italy
Data accessibility	Data are available in a public repository. Repository name: Mendeley Data Data identification number: 10.17632/m76d5rbtrd.1 (DOI) Direct URL to data: https://data.mendeley.com//datasets/m76d5rbtrd/1
Related Research Articles	The dataset shares part of the data described in the following papers: Marengo, D., Azucar, D., Longobardi, C., & Settanni, M. (2021). Mining Facebook data for Quality of Life assessment. *Behaviour & Information Technology*, *40*(6), 597-607. Marengo, D., Montag, C., Sindermann, C., Elhai, J. D., & Settanni, M. (2021). Examining the links between active Facebook use, received likes, self-esteem and happiness: A study using objective social media data. *Telematics and Informatics*, *58*, 101523. Marengo. D., Montag, C., Mignogna, A., Settanni, M. (2022, in press). Mining digital traces of Facebook activity for the prediction of individual differences in tendencies towards social networks use disorder: A machine learning approach. Frontiers in Psychology. Marengo, D., Poletti, I., & Settanni, M. (2020). The interplay between neuroticism, extraversion, and social media addiction in young adult Facebook users: Testing the mediating role of online activity using objective data. *Addictive behaviors*, *102*, 106150.

## Value of the Data

• This dataset can be useful to generate insights on the association between demographic variables, including age and gender, and personality (Big Five traits and Impulsivity/Sensation Seeking), and emotional expression as derived from social media pictures
• The dataset can be useful for researchers and data scientists who do research in social sciences, in particular psychoinformatics
• The dataset can be useful to train models in order to infer personality of users of social media platforms from profile pictures

## 1 Data Description

We retrieved the current profile picture of 2234 Italian Facebook users (596 men, 1,638 women; 73.7% in the 18–25 age group, and 15.5% in the 26–30 age group, 11.8% aged > 30) who also answered self-report questionnaires on demographic variables and personality. Data were collected between March and June 2018 using a Facebook application. Profile pictures consisting of 200 × 200 resolution jpegs were obtained by sending a request via the Facebook Graph API [1] and analyzed using online commercial services allowing for the scoring of facial expressions in image data, namely Microsoft Azure Face API [2] and MEGVII Face++ Detect API [3]**.** Both services provide emotional expression scores if at least one face is successfully detected in the picture. Using the Microsoft Azure Face API we obtained scores for anger, contempt, disgust, fear, joy, sadness, surprise, and neutrality. Using the MEGVII Face++ Detect API, pictures were scored for the presence of anger, disgust, fear, joy, sadness, and surprise, and neutrality. Higher scores on each emotion refer to a stronger expression of the respective emotion.

The dataset presented here [4] consists of data of N =728 Facebook users with a profile picture in which both APIs detected only one face (demographic characteristic: 171 men, 557 women; 74.6% in the 18–25 age group, and 15.9% in the 26–30 age group, 9.5% aged > 30). Data of users sharing a profile-picture in which at least one API failed to detect faces (N = 861), or at least one API detected multiple faces (N = 645) are not provided (hence 728 + 861 + 645 participants resulting in a total of the aforementioned 2234 participants). Additionally, since the sharing of Facebook profile pictures, although labeled as public data, violates the anonymity of participants, the data presented here only include emotional expression scores for the profile pictures, and participants’ scores on self-report questionnaires, while raw picture files are not provided.

Regarding self-report data, the dataset includes the following demographic information about the participants: gender and age. The dataset also includes participants’ personality scores based on short validated assessments of Big Five traits (Ten Item Personality Inventory, [5,6]), and Impulsivity/Sensation Seeking [7]. Please note that while all participants (N = 728) filled in demographics and the Big Five sections of the questionnaire, the Impulsivity/Sensation Seeking questionnaire was optional, resulting in a lower response rate on this questionnaire (N = 423).

Regarding the obtained emotional expression scores, please note that scores retrieved using the Microsoft Azure Face API scores are reported in their original metric, ranging from 0 to 1. In turn, the scores obtained using MEGVII Face++ Detect originally ranged from 0 to 100, but were rescaled to a 0-1 metric (i.e., a division by 100 was applied) for the purpose of comparability with Microsoft Azure Face API scores. In both cases, for each emotion scored by the APIs, higher scores indicate a stronger emotion intensity.

Table 1 provides mean emotional expressions scores for APIs, and illustrates gender differences emerging from the data using Mann-Whitney U tests. Happy and neutral expressions prevailed in the analyzed pictures according to both APIs; beyond that, males showed significantly more often a neutral facial expression than females, and for happiness the opposite pattern could be observed.

**Table 1:** Mean emotional expression scores obtained using Microsoft Azure Face and MEGVII Face++ Detect APIs in the overall sample and by gender group (N = 728).

	Total (N = 728)	Male (N = 171)	Female (N = 557)	p*
Microsoft Azure Face	Mean	Mean	Mean	
Anger	.0015	.0056	.0003	<.001
Contempt	.0084	.0152	.0063	<.001
Disgust	.0004	.0014	.0001	.007
Fear	.0011	.0006	.0013	.020
Happiness	.5859	.3928	.6452	<.001
Neutral	.3879	.5691	.3323	<.001
Sadness	.0065	.0062	.0066	.033
Surprise	.0080	.0090	.0077	.247

MEGVII Face++ Detect	Mean	Mean	Mean	

Anger	.0223	.0320	.0193	.037
Disgust	.0213	.0212	.0213	.039
Fear	.0251	.0277	.0243	.929
Happiness	.5181	.3329	.5749	<.001
Neutral	.3103	.4890	.2554	<.001
Sadness	.0488	.0392	.0517	.968
Surprise	.0542	.0579	.0531	.373

*Note:* * p values for Mann-Whitey U tests for between-gender differences.

Table 2 illustrates Spearman correlations computed between the emotional expression scores obtained using the two APIs. Strong correlations are observed between happiness and neutrality scores as obtained from the two APIs, while concordance of scores for other emotions is generally small-to-moderate. The APIs came largely to similar conclusions when judging upon the happy or neutral facial expressions. The overlap when analyzing the remaining facial expressions was mostly in the low effect size area.

**Table 2:** Spearman correlations between emotional expression scores obtained using Microsoft Azure Face and MEGVII Face++ Detect APIs (N = 728).

	MEGVII Face++ Detect						
Microsoft Azure Face	Anger	Disgust	Fear	Happiness	Neutral	Sadness	Surprise
Anger	.23*	.16*	.15*	-.23*	.16*	.13*	.13*
Contempt	.28*	.23*	.26*	-.36*	.40*	.30*	.34*
Disgust	.14*	.16*	.16*	-.11*	.01	.11*	.15*
Fear	.11*	.13*	.20*	-.19*	.05	.15*	.16*
Happiness	-.33*	-.20*	-.28*	.85*	-.80*	-.34*	-.41*
Neutral	.32*	.19*	.26*	-.82*	.81*	.31*	.39*
Sadness	.27*	.16*	.25*	-.60*	.49*	.36*	.27*
Surprise	.19*	.17*	.24*	-.31*	.18*	.15*	.33*

*Note:* * p <.05. Please note that a score for contempt is only returned by the Microsoft Azure Face API.

Table 3 provides descriptive statistics for self-reported measures of personality.

**Table 3:** Mean, standard deviation, minimum and maximum values of self-report personality scores.

	N	Mean	Std. Deviation	Min	Max
Big Five					
Extraversion	728	3.97	1.64	1.00	7.00
Agreeableness	728	5.30	1.14	1.00	7.00
Coscientiousness	728	5.20	1.27	1.50	7.00
Emotional stability	728	3.89	1.44	1.00	7.00
Openness	728	5.09	1.13	1.00	7.00
Impulsivity/Sensation Seeking	423	3.17	2.01	0.00	8.00

**Fig. 1:**
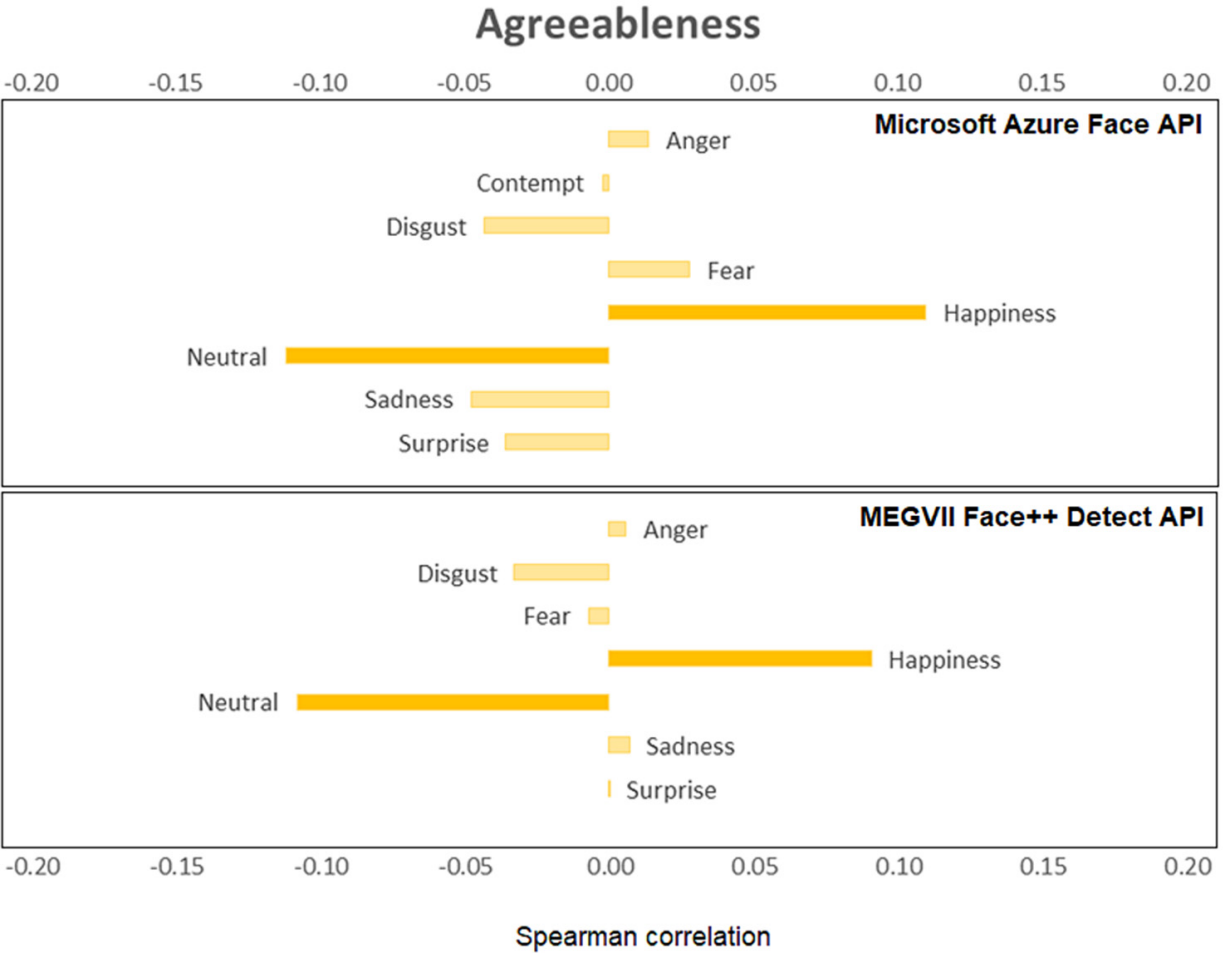
Spearman correlations between emotional expression scores and agreeableness.

**Fig. 2:**
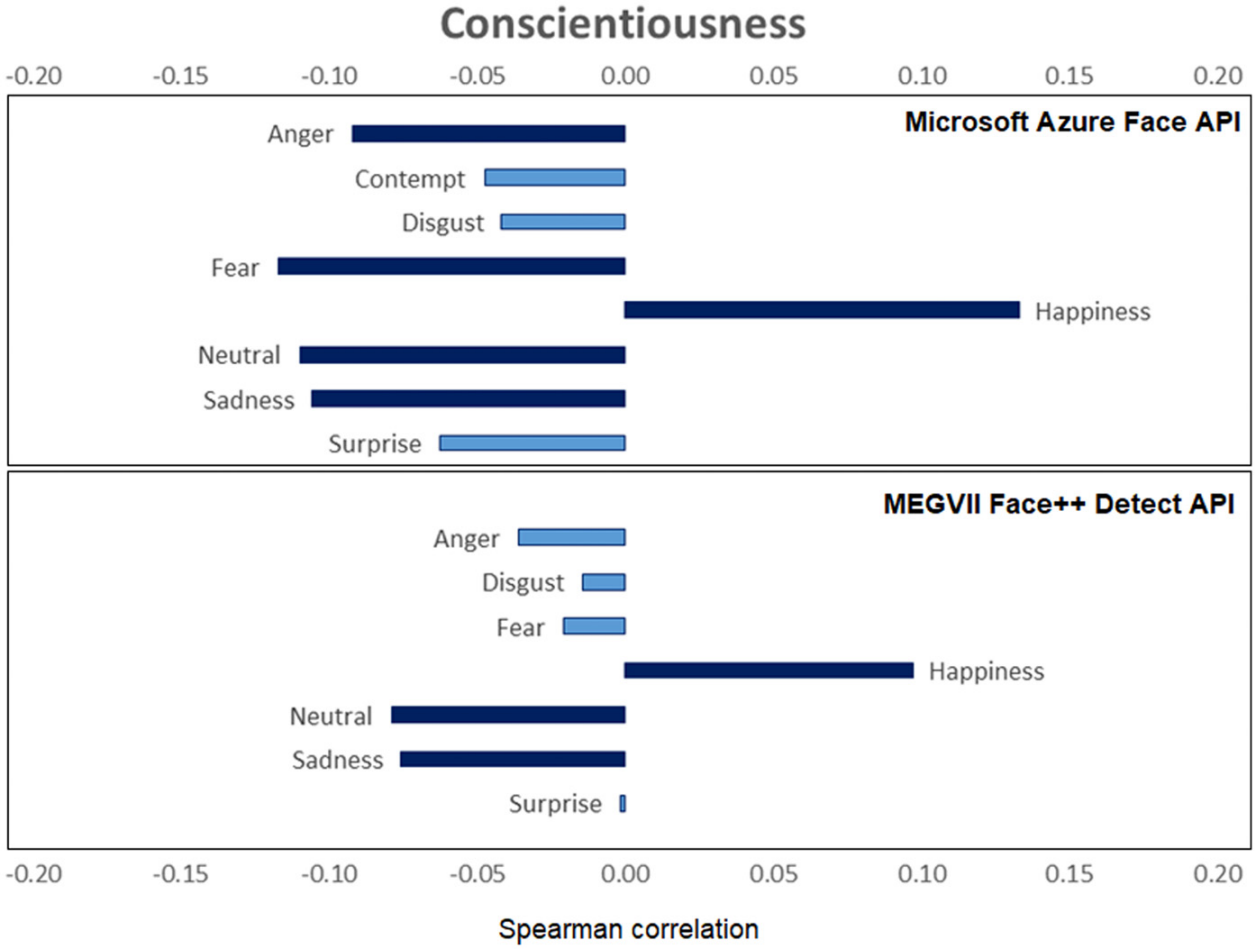
Spearman correlations between emotional expression scores and conscientiousness.

**Fig. 3:**
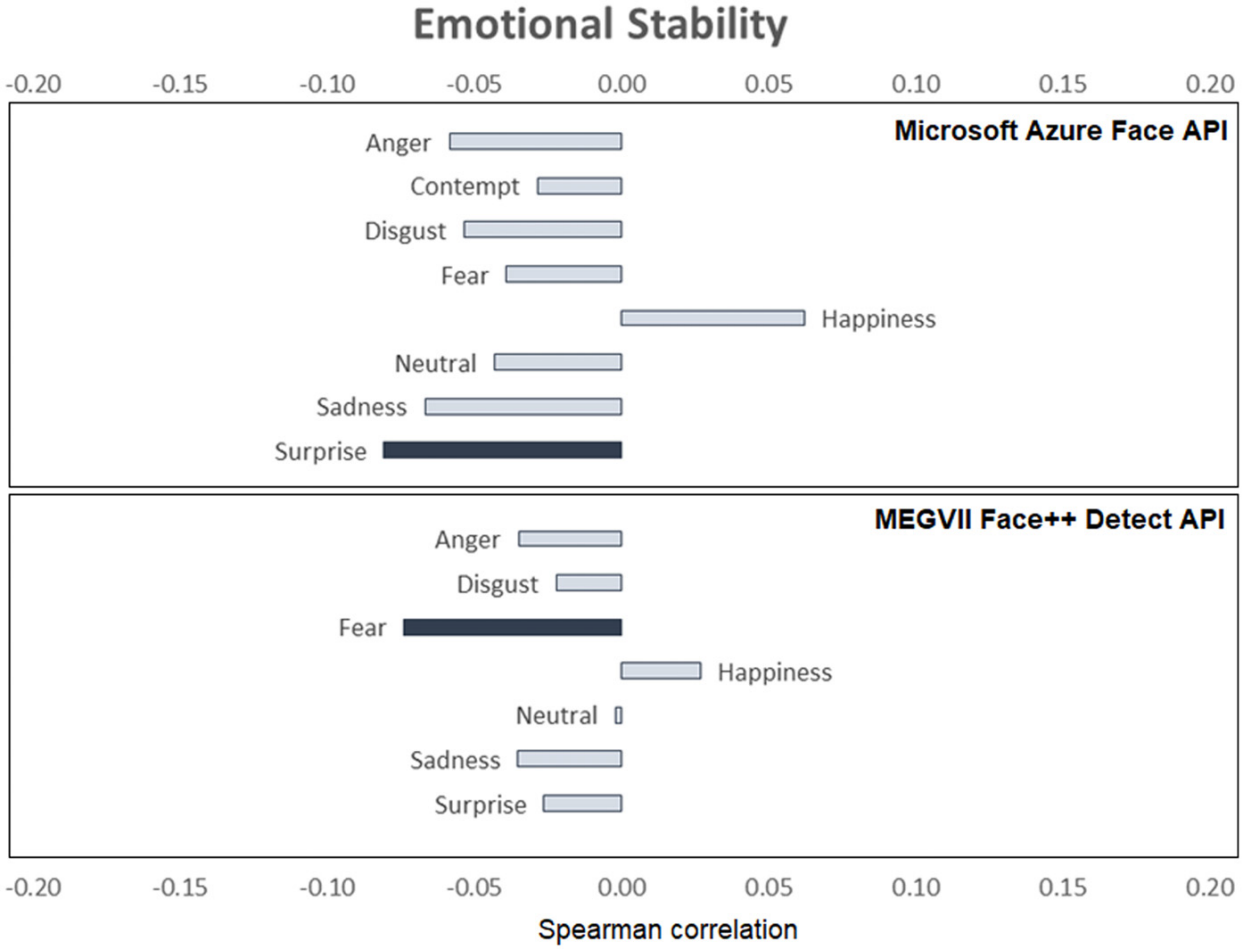
Spearman correlations between emotional expression scores and emotional stability.

**Fig. 4:**
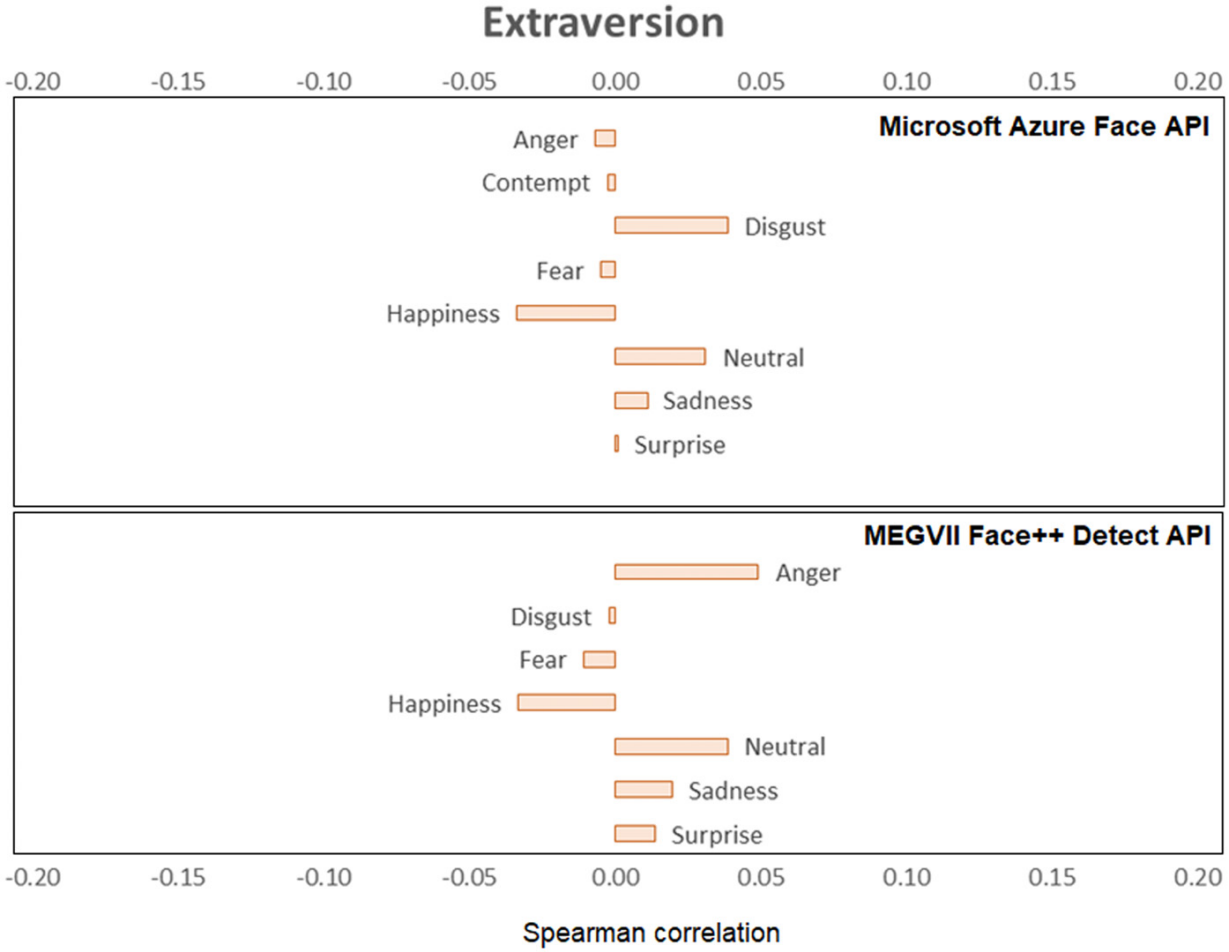
Spearman correlations between emotional expression scores and extraversion.

**Fig. 5:**
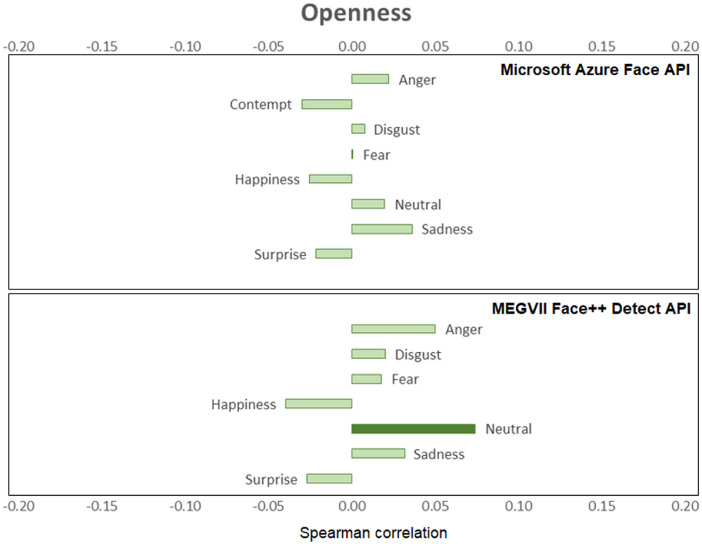
Spearman correlations between emotional expression scores and openness.

**Fig. 6:**
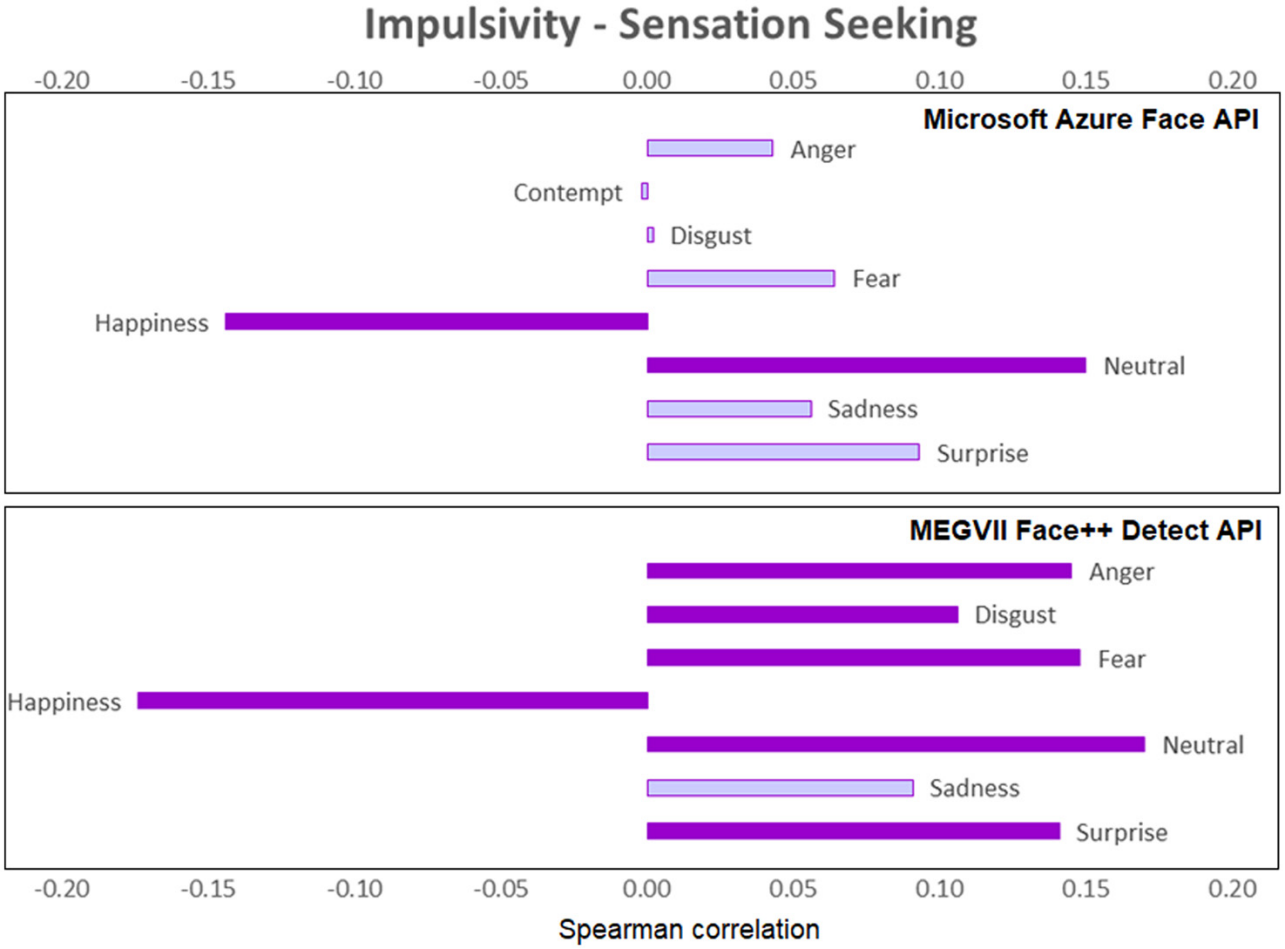
Spearman correlations between emotional expression scores and impulsivity/sensation seeking.

Fig. 1, Fig. 2, Fig. 3, Fig. 4, Fig. 5, Fig. 6 provide a visualization of Spearman correlations computed between the emotional expression scores obtained using the two APIs, and participants’ personality scores. Note that in these figures, bars rendered with a darker shade indicate a significant (p<.05) correlation. All personality traits showed at least one significant correlation with emotional expression scores, except for extraversion. Agreeable persons tend to show more happy and less neutral faces (but effect sizes are generally low). Conscientious persons tend to show more happy faces and less neutral/sadness faces, and differences across APIs can be observed in the anger/fear-conscientiousness associations. Correlations between emotional expression scores and extraversion, emotional stability, and openness are more inconclusive. Finally, persons that are more impulsive tend to show more neutral and less happy facial expressions, and this is true for both APIs, while association with other emotional expressions tend to differ across APIs.

## 2 Experimental Design, Materials and Methods

We recruited adult participants by disseminating online the link to a Facebook web application allowing for both the administration of an online survey and the collection of users’ digital traces on the Facebook platform. The administered app was designed by two of the authors (DM, MS) as a web application external to Facebook, and implemented by two research assistants (see acknowledgements) using PHP for the server logic and JavaScript/jQuery for the dynamic parts of the pages administering questionnaires. The landing page of the web application included a Facebook login collecting participants’ consent so that Facebook profile data could be shared with the researchers; the page also included an informed consent form for participation in the online survey. Next, participants were presented with an online survey including questions regarding demographic variables (gender, age) and self-report questionnaires assessing personality traits, including Big Five traits [5,6] and impulsivity/sensation seeking [7]. The English translation of administered questionnaires relevant to the data presented here is provided along with the dataset as supplementary material. Please note that a partial overlap exists in the present dataset with data examined in other published studies [[8], [9], [10], [14]], although this overlap only pertains to self-reported demographics and/or personality data.

The app was initially disseminated online by a seed of 10 university students attending Master's Degree classes in Psychology at University of Turin, Italy. Data collection took place from March to June 2018. In order to be enrolled in the study, participants had to be active Facebook users, needed to be at least 18 years old, and provided access to their Facebook profile data using the Facebook login.

Eventually, public profile pictures of participants were retrieved through the Facebook Graph API by using the getUsers() function of the Rfacebook package for R [11]. Emotional expression scores for the retrieved images were obtained by sending request calls to the Microsoft Azure Face API [2] and the MEGVII Face++ Detect API [3]. The code used for performing the API calls was adapted from the code provided by Theresa Küntzler (University of Konstanz) on GitHub [12] as used in a previous publication (e.g. [13]).

## Ethics Statements

All participants provided researchers with informed consent to collect public Facebook profile data, as well as posts, and Likes data. Please note that the dataset presented here does not include raw Facebook picture data. Data have been fully anonymized and complies with current Facebook data redistribution policies. The research was approved by the university institutional review board at University of Turin, Italy (n° 88721).

## CRediT author statement

**Davide Marengo:** Conceptualization, Methodology, Software, Data curation, Writing – Original draft preparation; **Michele Settanni**: Conceptualization, Methodology, Supervision, Project administration, Funding Acquisition; **Christian Montag:** Validation, Writing – Reviewing/Revising, Interpretation and Editing.

## Declaration of Competing Interest

The authors declare that they have no known competing financial interests or personal relationships that could have appeared to influence the work reported in this paper.

The authors declare the following financial interests/personal relationships which may be considered as potential competing interests:

CM mentions that he has received (to Ulm University and earlier University of Bonn) grants from agencies such as the German Research Foundation (DFG). CM has performed grant reviews for several agencies; has edited journal sections and articles; has given academic lectures in clinical or scientific venues or companies; and has generated books or book chapters for publishers of mental health texts. For some of these activities he received royalties, but never from gaming or social media companies. CM mentions that he is part of a discussion circle (Digitalität und Verantwortung: https://about.fb.com/de/news/h/gespraechskreis-digitalitaet-und-verantwortung/) debating ethical questions linked to social media, digitalization and society/democracy at Facebook. In this context, he receives no salary for his activities. Finally, he mentions that he currently functions as independent scientist on the scientific advisory board of the Nymphenburg group. This activity is financially compensated.
